# HBV replication is significantly reduced by IL-6

**DOI:** 10.1186/1423-0127-16-41

**Published:** 2009-04-20

**Authors:** Tzer-Min Kuo, Cheng-po Hu, Ya-Ling Chen, Ming-Hsiang Hong, King-Song Jeng, Chun-Chin T Liang, Mong-Liang Chen, Chungming Chang

**Affiliations:** 1 Division of Molecular and Genomic Medicine, National Health Research Institutes, Miaoli, Taiwan, ROC; 2 Institute of Microbiology and Immunology, National Yang-Ming University, Taipei, Taiwan, ROC; 3 Department of life Science, Tunghai University, Taichung, Taiwan, ROC; 4 Institute of Molecular Biology, Academia Sinica, Taipei, Taiwan, ROC; 5 Institute of Cellular and System Medicine, National Health Research Institutes, Miaoli, Taiwan, ROC; 6 Department of General Education, National Taipei College of Nursing, Taipei, Taiwan, ROC

## Abstract

Interleukin-6 (IL-6) is a pleiotropic cytokine with pivotal functions in the regulation of the biological responses of several target cells including hepatocytes. The level of serum IL-6 has been reported to be elevated in patients with chronic hepatitis B, cirrhosis and hepatocellular carcinoma and represents the best marker of HBV-related clinical progression as compared with several other cytokines. In this study, we found that IL-6 was able to effectively suppress hepatitis B virus (HBV) replication and prevent the accumulation of HBV covalently closed circular DNA (cccDNA) in a human hepatoma cell line. We also demonstrated that the suppression of HBV replication by IL-6 requires concurrently a moderate reduction of viral transcripts/core proteins and a marked decrease in viral genome-containing nucleocapsids. Studies on the stability of existing viral capsids suggest that the IL-6 effect on the reduction of genome-containing nucleocapsids is mediated through the prevention of the formation of genome-containing nucleocapsids, which is similar to the effect of interferons. However, IFN-α/β and IFN-γ did not participate in the IL-6-induced suppression of HBV replication. Taken together, our results will provide important information to better understand the role of IL-6 in the course of HBV infection.

## Background

Hepatitis B virus (HBV) is a hepatotropic, non-cytopathic DNA virus (3.2 kb partially double-stranded DNA) that causes acute and chronic hepatitis. More than 350 million people worldwide suffer from chronic hepatitis B (CHB) infection, which is associated with a high risk of developing cirrhosis and hepatocellular carcinoma [1,2]. The interactions between HBV replication and immune responses against HBV infection play an important role in determining the outcome of virus infection [3,4]. Previous studies using chimpanzees and transgenic mice models have indicated that HBV clearance occurs prior to the destruction of infected cells [5,6]. These results suggest that cytokines are likely to be involved in both the regulation of the immune responses and the direct inhibition of HBV replication. Several cytokines have recently been shown to effectively suppress HBV replication in a noncytopathic manner in HBV transgenic mice and in a cell culture system. Interleukin-12 (IL-12), IL-18 and intrahepatic induction of alpha/beta interferon (IFN-α/β) are able to effectively inhibit HBV replication in the liver of transgenic mice [7-9]. IFN-α/β, gamma interferon (IFN-γ) and tumor necrosis factor alpha (TNF-α) suppress HBV replication in immortalized murine hepatocytes and human hepatoma cells by preventing the formation of viral capsids or disrupting capsid integrity [10,11]. Furthermore, IL-4 and transforming growth factor beta-1 (TGF-β1) have been demonstrated to suppress HBV replication in hepatoma cells through the transcriptional regulation of HBV RNA [12,13]. These studies suggest that inflammatory cytokines play an important role in the antiviral response against HBV infection.

IL-6 is one of the major inflammatory cytokines, and in several types of target cells it affects a variety of biological responses including changes in cell differentiation, growth, apoptosis and the induction of acute-phase responses [14,15]. In response to liver injury, IL-6 expression is induced in various cell types including endothelial cells, hepatocytes and Kupffer cells [16]. IL-6 plays an important role in promoting hepatic survival by stimulating liver regeneration, and protects the liver from damage caused by immune responses, alcohol and viral infection. The level of serum IL-6 has been reported to be elevated in patients with CHB, cirrhosis and hepatocellular carcinoma, relative to normal subjects [17-19]. IL-6 activity has been shown to be significantly enhanced during acute exacerbation of CHB, which is accompanied by clearance of HBV e antigen (HBeAg). Interestingly, the level of serum IL-6 were reported to be inversely correlated to the transaminase level in patients and represents the best marker of HBV-related clinical progression as compared with IL-10, IL-12 and IFN-γ [20]. Recent experiments have also indicated that gender may influence MyD88-dependent IL-6 production by Kupffer cells, and this may contribute to gender disparity in hepatocarcinogenesis [21]. Using a human-mouse radiation chimera model, Galun et al. found that IL-6 could facilitate HBV infection and suggested that IL-6 might be a potential mediator for HBV entrance into hepatocytes [22]. However, the effect and the mechanisms of action of IL-6 on HBV replication have not been studied in detail.

In this study, we found that IL-6 can effectively suppress HBV replication in an HBV-producing cell line, 1.3ES2 [23]. The suppression of HBV replication requires a moderate reduction of viral transcripts/core proteins and a marked decrease in the formation of HBV genome-containing nucleocapsids. Our studies provide important information to reveal the role of IL-6 in the course of HBV infection.

## Materials and methods

### Cell culture

HepG2 and 1.3ES2 cells were maintained in Dulbecco's modified Eagle's medium (DMEM) containing 10% fetal calf serum, as described previously [23]. After culture for 4 days, the confluent 1.3ES2 cells were serum-deprived for 2 days and treated with human IL-6 (R&D Systems Inc., Minneapolis, USA) for various periods to assess the antiviral effect of IL-6. The culture medium was refreshed every 2 days during the experiments. For the neutralization experiment, sheep polyclonal anti-IFN-β antibody (PBL Biomedical Laboratories, New Jersey, USA) was added to the culture medium to block the endogenous IFN-β activity. Human IFN-β (PBL Biomedical Laboratories, New Jersey, USA) was added as a control for the neutralization experiment.

### Southern blot analysis

Total DNA (20 μg) was digested with HindIII and separated on 0.7% agarose gels. The gels were treated as described previously [23] and the DNA samples were transferred onto nylon membranes (Amersham, Freiburg, Germany). After ultraviolet crosslinking and prehybridization, the membranes were hybridized with an HBV-specific probe generated using a random-primed labeling kit (Amersham, Freiburg, Germany).

### Analysis of intracellular HBV genome-containing nucleocapsid

Analysis of intracellular HBV genome-containing nucleocapsids was performed as described previously [13,24]. Briefly, cell lysates were separated using a 0.8% native agarose gel and transferred onto nylon membranes. Capsid-associated nucleic acids were released from the nucleocapsids in situ by denaturing with 0.2 N NaOH/1.5 M NaCl followed by neutralization with 0.2 N Tris-HCl/1.5 M NaCl. The membranes were then hybridized with an HBV-specific probe.

### Northern blot analysis

Total RNA was isolated using TRIzol solution (Invitrogen, California, USA) according to the manufacturer's instructions, and 15 μg of isolated RNA was separated using a 1.2% formaldehyde-agarose gel and transferred onto nylon membrane. The membranes were then hybridized with an HBV-specific probe.

### Western blot analysis

Total protein (100 μg) was separated using a 15% SDS-polyacrylamide gel and then transferred onto polyvinylidene fluoride membranes (Millipore, Massachusette, USA). The membranes were incubated with anti-core antibody (DakoCytomation, Glostrup, Denmark) or anti-actin antibody (Sigma, St. Louis, MO). The immunoblot signals were examined using enhanced chemiluminescence reagent (Millipore, Massachusette, USA).

### Analysis of extracellular HBV nucleic acids

The extracellular pool of HBV nucleic acids was extracted from cell culture medium using a High Pure Nucleic Acid Kit (Roche Applied Science, Mannheim, Germany). The level of viral genomes was then measured by real-time PCR method using a LightCycler^® ^TaqMan^® ^Master Kit (Roche Applied Science, Mannheim, Germany) with a pair of HBV-specific primer (forward primer: 5'-GCTCCAGTTCAGGAACAGTAAAC-3' and reverse primer: 5'-AATCCTCGAGAAGATTGACGAT-3') and the Taqman probe #34. The thermo-cycling parameters were 94°C for 10 min; 50 cycles of 94°C for 10 sec, 60°C for 30 sec, and 72°C for 1 min; and 40°C for 30 sec.

### Detection of HBsAg and HBeAg

The levels of HBV surface antigen (HBsAg) and HBeAg in the culture medium were assessed using an enzyme-linked immunosorbent assay (ELISA) following the manufacturer's protocol (Evernew Biotech Inc, Taipei, Taiwan). Each experiment was performed in triplicate and independently repeated three times.

### Nucleocapsid stability

Confluent 1.3ES2 cells were labeled with [^35^S] methionine-cysteine protein labeling mix (Perkin-Elmer, Connecticut, USA) for 6 h and then chased by replacing with unlabeled medium in the absence or presence of IL-6 (20 ng/ml or 40 ng/ml) for 24 h or 48 h. Cell lysates were harvested and the nucleocapsids were separated from free core protein by centrifugation through a Centricon-100 filter with a retention cutoff of 100 kDa (Millipore, Massachusette, USA). Total core proteins and nucleocapsids were then immunoprecipitated using an anti-core antibody and separated by 12% SDS-polyacrylamide gel electrophoresis. The signal of labeled core protein was visualized by autoradiography.

### Hirt extraction

HBV cccDNAs were isolated as previously described [23]. After complete lysis of the cells with Hirt solution, 750 μl of 5 N NaCl was added to the cell lysate and the mixture incubated on ice overnight. The supernatant was collected after centrifugation and extracted with phenol/chloroform. HBV cccDNA was then precipitated by ethanol and examined by Southern blot analysis.

### Primer pairs for RT-PCR

The cDNA templates were amplified by the following primer pairs: (1) IFN-α2b forward primer: 5'-TCCTAGACAAATTCTACACTGAAC-3', IFN-α2b reverse primer: 5'-GCTCTGACAACCTCCC-3'. (2) IFN-β forward primer: 5'-AAACTCATGAGCAGTCTGCA-3', IFN-β reverse primer: 5'-AGGAGATCTTCAGTTTCGGAGG-3'. (3) IFN-γ forward primer: 5'-TCAGCTCTGCATCGTTTTGG-3', IFN-γ reverse primer: 5'-GTTCCATTATCCGCTACATCTGAA-3'. (4) β-actin forward primer: 5'-TGAACTGGCTGACTGCTGTG-3', β-actin reverse primer: 5'-CATCCTTGGCCTCAGCATAG-3'. (5) GBP-1 forward primer: 5'-ACAAGGGAACAGCCTGGACATGG-3', GBP-1 reverse primer: 5'-GCCCACAATTGCCACCACCA-3'. (6) MxA forward primer: 5'-ACCACAGAGGCTCTCAGCAT-3', MxA reverse primer: 5'-CTCAGCTGGTCCTGGATCTC-3'. (7) GAPDH forward primer: 5'-GCTGAGAACGGGAAGC-3', GAPDH reverse primer: 5'-GGTGAAGACGCCAGTG-3'.

## Results

### IL-6 suppressed HBV replication in the HBV-producing cell line 1.3ES2

**Figure 1:**
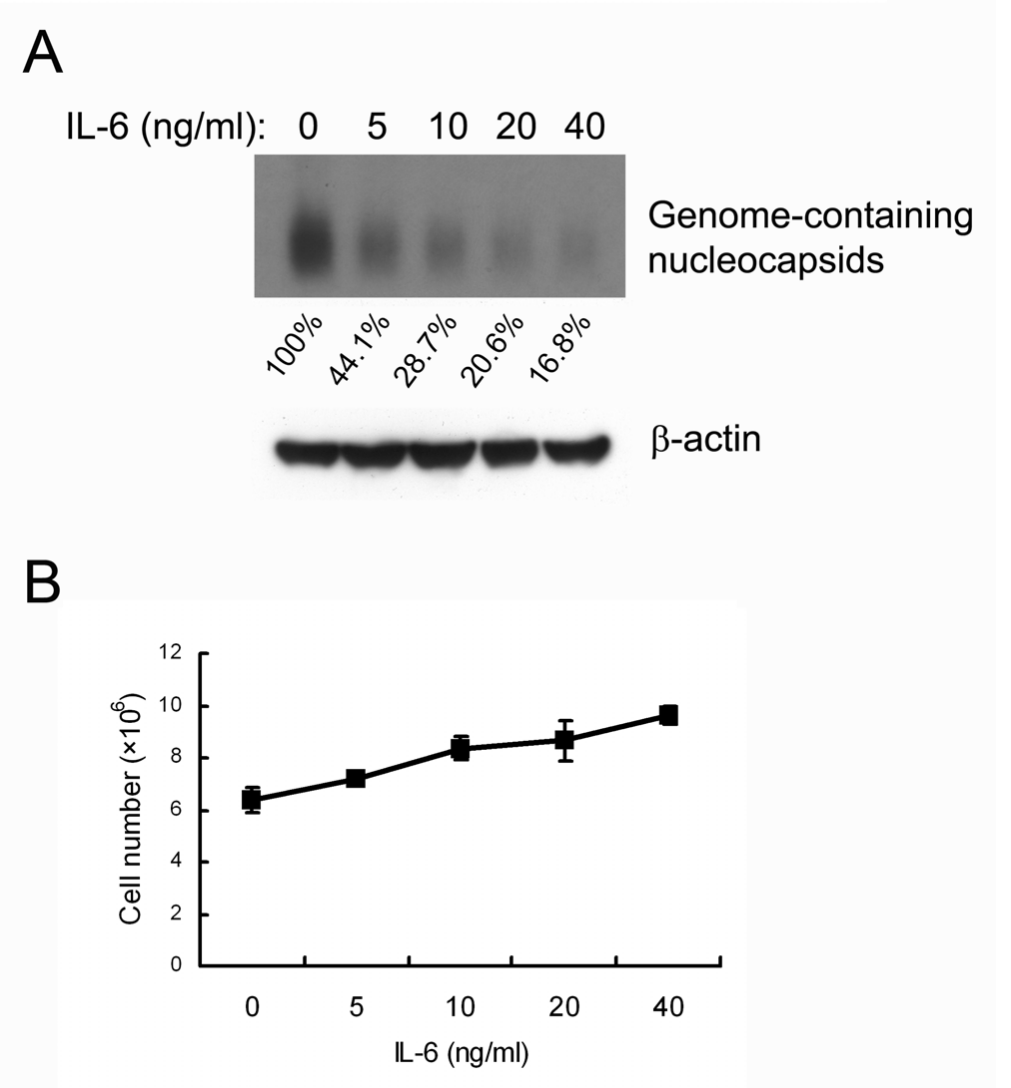
**IL-6 suppresses HBV replication in a dose-dependent manner**. (A) 1.3ES2 cells were treated 6 days after plating (when the cells had reached confluence) with various doses of IL-6 (0, 5, 10, 20, 40 ng/ml). The cell lysates were harvested after 4 days treatment with IL-6 and equal amounts of sample were analyzed by particle blot analysis using native agarose gel electrophoresis. HBV genome-containing nucleocapsids were detected by Southern blot analysis of the disrupted nucleocapsids, using an HBV-specific probe. Expression of β-actin was used as an internal control for sample loading. The signals were quantified by densitometry analysis and expressed as percentage of the control cells to indicate the inhibitory effect of IL-6. (B) Effect of IL-6 on growth curve. The cells were treated with IL-6, as described above. The cell number was determined by the trypan blue exclusion method.

To evaluate the effect of IL-6 on HBV replication, an HBV-producing cell line (1.3ES2) was established by stably transfecting HepG2 cells with 1.3-fold HBV genome [23]. The 1.3ES2 cells were chosen because of their abundant expression of viral replicative intermediates and the capacity to support the formation of covalently closed circular DNA (cccDNA). To determine the dose of IL-6 that effectively interfered with HBV replication, confluent 1.3ES2 cells (day 6 after plating) were treated with various concentrations of IL-6 under serum-starvation conditions. For the detection of intracellular HBV genome-containing nucleocapsids, cell lysates were separated by native agarose gel electrophoresis and performed by particle blot analysis. The viral genome-containing nucleocapsids were detected with an HBV-specific probe. After treatment for 4 days, IL-6 reduced HBV genome-containing nucleocapsids in a dose-dependent manner, with significant suppression effect observed at a concentration of 5 ng/ml (Fig. 1A). At 20 ng/ml of IL-6, a marked reduction (79.4%) of genome-containing nucleocapsids was evident as compared with the mock control. The effect of IL-6 on the proliferation of 1.3ES2 cells was also examined. The results indicated that growth of 1.3ES2 cells under serum-starvation increased in a dose-dependent manner after 4 days of treatment (Fig. 1B). There was no obvious apoptotic cell death in either the IL-6-treated or the control groups (data not shown). These results indicate that although IL-6 slightly increased 1.3ES2 cell proliferation, it could effectively inhibit HBV replication in a noncytopathical manner.

**Figure 2:**
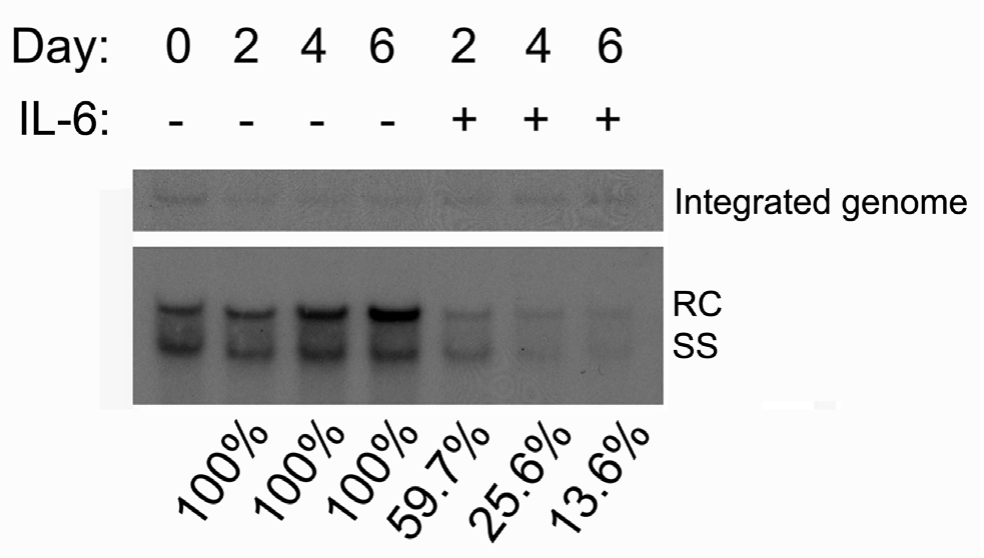
**IL-6 suppresses HBV replication in a time-dependent manner**. Confluent cells (day0) were treated with or without 20 ng/ml of IL-6 for 2 days (day2), 4 days (day4) or 6 days (day6). For the analysis of HBV replicative intermediates, equal amounts of total DNA were subjected to HindIII digestion and separated by electrophoresis. The integrated viral genome and viral replicative intermediates were then examined by Southern blot analysis using an HBV-specific probe. Bands corresponding to the integrated genome, the relaxed circular double-stranded DNA (RC) and single-stranded DNA (SS) are indicated individually. The intensity of the integrated HBV genome in each lane reflected the amounts of total DNA and was served as the internal control for sample loading. The signals were quantified by densitometry analysis and expressed as percentage of the respective control cells to indicate the inhibitory effect of IL-6.

To investigate the kinetics of IL-6 on suppression of HBV replication, confluent cells (indicated as day 0) were treated with 20 ng/ml of IL-6, and total cellular DNA was extracted at day 2, day 4 and day 6. The levels of HBV replicative intermediates increased with time in the control cells, but decreased dramatically in the IL-6-treated cells (Fig. 2). At day 2, the level of DNA replicative intermediates in IL-6-treated cells was reduced to 59.7% of the control cells, at day 4 was reduced to 25.6%, and at day 6 was reduced to 13.6%. These results indicate that HBV replication was significantly suppressed by IL-6 treatment in a time-dependent manner.

### IL-6 suppressed HBV replication through moderate reduction of viral transcripts and marked decrease in the genome-containing nucleocapsid levels

**Figure 3:**
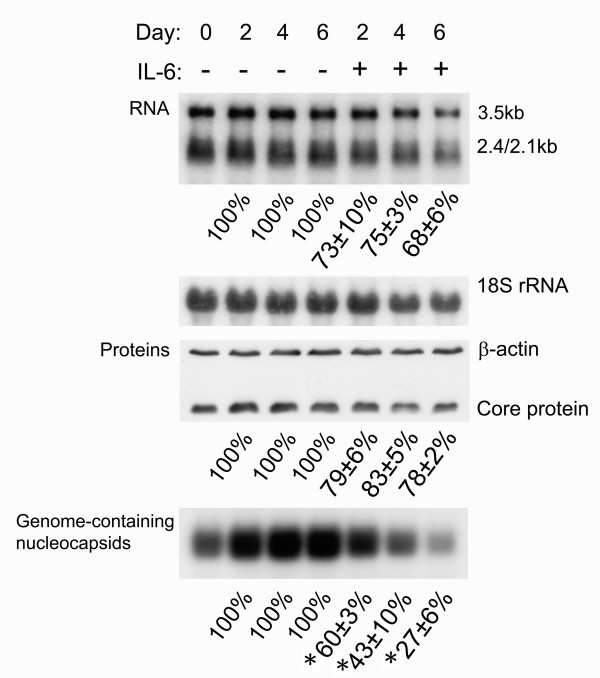
**IL-6 decreases the levels of HBV transcripts, core protein, and genome-containing nucleocapsids**. Confluent cells (day0) were treated with or without 20 ng/ml of IL-6 for 2 days (day2), 4 days (day4) or 6 days (day6). For RNA analysis (panel for RNA), total RNAs were extracted and equal amounts of samples were subjected to Northern blot analysis to reveal the expression profile of the HBV transcripts, namely those of 3.5-kb and 2.4-kb/2.1-kb respectively. The expression level of 18S rRNA was used as an internal control for sample loading. For Western blot analysis (panel for Proteins), cell lysates were harvested and equal amounts of samples were separated by SDS-polyacrylamide gel electrophoresis. The total core protein was subsequently detected by immunoblot analysis with antibodies against core protein. The expression level of β-actin was used as an internal control for sample loading. For particle blot analysis, equal amounts of cell lysates were assayed for viral capsid by native agarose gel electrophoresis. HBV genome-containing nucleocapsids were then detected by Southern blot analysis of the disrupted nucleocapsids using an HBV-specific probe (panel for Genome-containing nucleocapsids). The signals including 3.5-kb RNA, core proteins and genome-containing nucleocapsids were quantified by densitometry analysis and expressed as the average percentage of respective control cells from three independent experiments to indicate the inhibitory effect of IL-6. Results shown are representative of three independent experiments. *: The reduction in levels of HBV genome-containing nucleocapsids was compared with that of core proteins and found to be highly significant (P < 0.05 as monitored by Pearson χ^2 ^test).

To further characterize the mechanisms involved in the antiviral effect of IL-6, the levels of viral transcripts, core protein and genome-containing nucleocapsids were simultaneously examined with or without IL-6 treatment in three independent experiments. A representative experiment and the statistical analyses are given in Fig. 3. After IL-6 treatment, the HBV transcripts and core protein were moderately reduced (Fig. 3, panels for RNA and proteins). At day 2 and day 4 the levels of HBV RNAs were reduced in IL-6 treated cells to 73 ± 10% and 75 ± 3% as compared with the control cells, and at day 6 was reduced to 68 ± 6%. Similarily, the levels of core protein were also reduced by IL-6. At day 2, day 4 and day 6, the levels of core protein were reduced to 79 ± 6%, 83 ± 5% and 78 ± 2% as compared with the control cells. Interestingly, the genome-containing nucleocapsids were decreased to more significant levels after IL-6 treatment (Fig. 3, panel for genome-containing nucleocapsids). At day 2, the level of genome-containing nucleocapsids was reduced in IL-6 treated cells to 60 ± 3% of control cells, at day 4 was reduced to 43 ± 10% and at day 6 was reduced to 27 ± 6%. The reduction in levels of HBV genome-containing nucleocapsids was compared to that of core proteins and the differences were highly significant (p = 0.03, 0.00 and 0.01 at day 2, 4 and 6 respectively). These results clearly indicated that the dramatically inhibitory effect on HBV genome-containing nucleocapsids can not be explained only by the moderate reduction of HBV transcripts and core proteins. It is likely that the dramatic reduction of genome-containing nucleocapsids also involves the disruption of existing capsids, which is similar to the effect of TNF-α [11], or interference with the assembly of genome-containing nucleocapsids as interferons [10].

**Figure 4:**
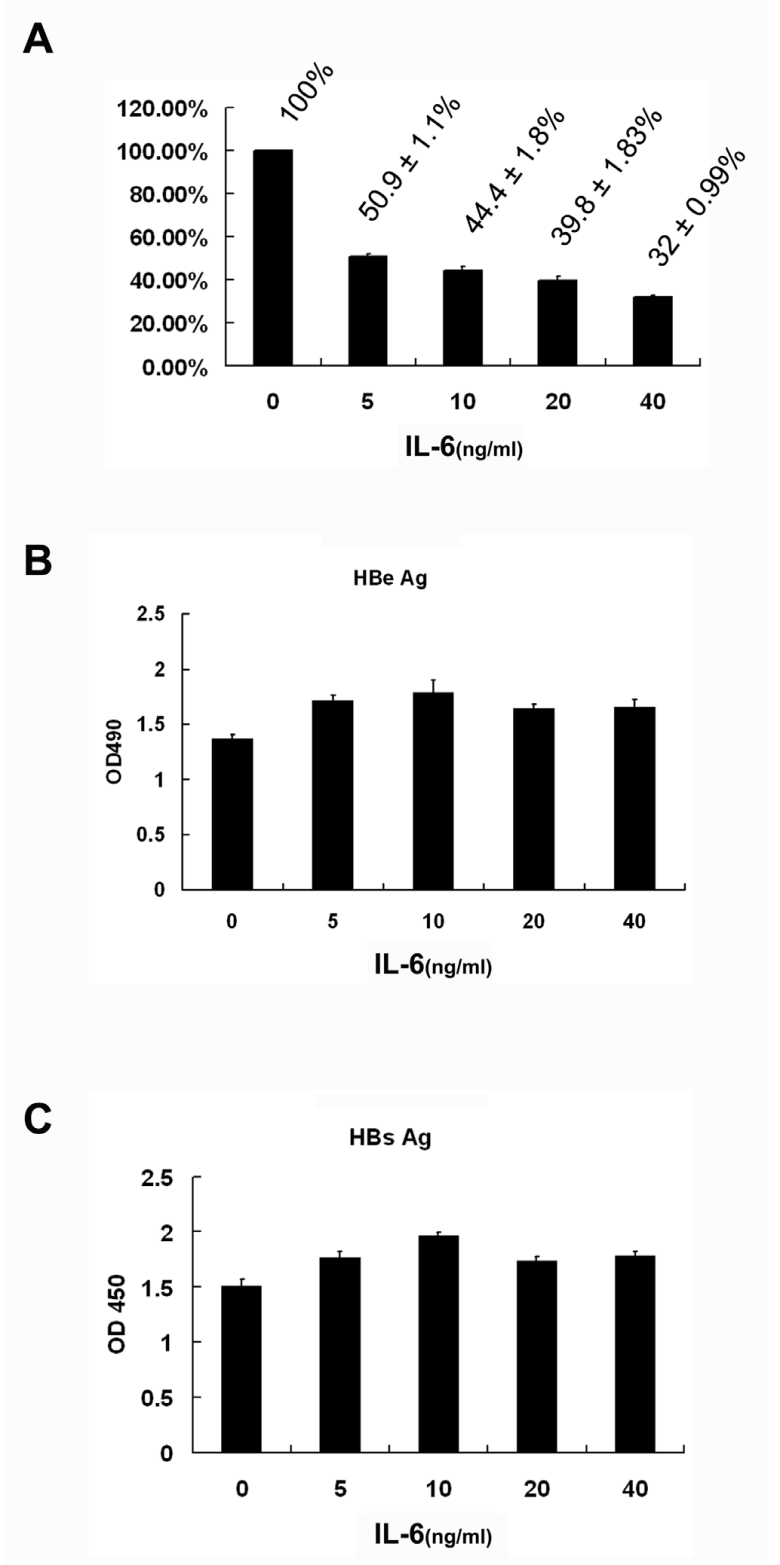
**IL-6 does not suppress the levels of secreted HBeAg and HBsAg**. Confluent cells were treated for 6 days with various doses of IL-6 (0, 5, 10, 20, 40 ng/ml). (A) The level of extracellular viral genome-containing nucleocapsids in the culture medium was measured by real-time PCR method and normalized with total cell number. (B and C) The levels of secreted HBeAg and HBsAg in the culture medium were measured by ELISA. Results shown are representative of three independent experiments.

To assess whether IL-6 could also affect the extracellular pool of viral genome-containing nucleocapsids in the culture medium, the level of secreted viral genome in the presence of different concentrations of IL-6 was estimated by real-time PCR method. As shown in Figure 4A, the level of viral genomes was dramatically decreased with IL-6 treatment for 6 days in a dose-dependent manner with significant reduction observed at 5 ng/ml. At 20 ng/ml of IL-6, a marked reduction (39.8%) of genome-containing nucleocapsids was evident as compared with the mock control. However, the quantities of secreted HBeAg and HBsAg in the culture medium were not decreased by IL-6 treatment as measured by ELISA analysis (Fig. 4B and 4C). Taken together, these results imply that IL-6 exerts its antiviral activity through combinational effects which include a moderate reduction of HBV transcripts/core proteins and a marked decrease in HBV genome-containing nucleocapsids.

### IL-6 did not disrupt the HBV nucleocapsids

**Figure 5:**
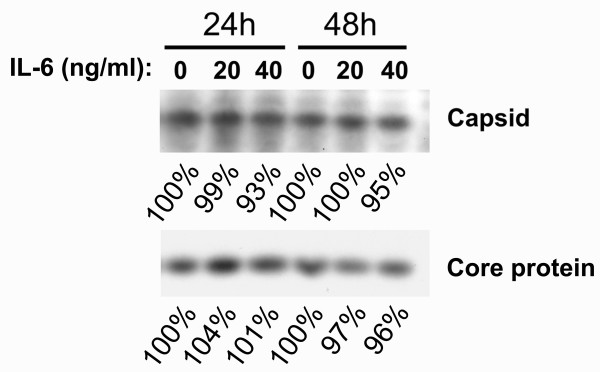
**IL-6 does not disrupt the HBV nucleocapsids**. To assess the IL-6 effect on the stability of HBV nucleocapsids, confluent cells were labeled with [^35^S] methionine-cysteine protein labeling mix for 6 h and then chased in the absence or presence of IL-6 (20 ng/ml or 40 ng/ml) for 24 h or 48 h. Cell lysates were harvested and the nucleocapsids were separated from free core protein by centrifugation through a Centricon-100 filter with a retention cutoff of 100 kDa. Total core protein and nucleocapsids were then immunoprecipitated using an anti-core antibody and separated by SDS-polyacrylamide gel electrophoresis. The labeled core protein was visualized by autoradiography. The signals were quantified by densitometry analysis and expressed as percentage of the respective control cells to indicate the IL-6 effect on capsid stability. Results shown are representative of three independent experiments.

To understand whether the disruption of existing nucleocapsids is involved in the suppression of HBV replication, 1.3ES2 cells were labeled with [^35^S] methionine-cysteine for 6 h and treated with IL-6 for 24 h or 48 h. Following lysis, total cell lysates were collected. The intact core particles were collected by centrifugation through centricone filter with cutoff of 100 kDa as reported previously [11]. The total lysate and the intact core particles were then immunoprecipitated with anti-core antibody. As shown in Fig. 5, there was no appreciable change in the levels of total HBV core proteins or viral capsids after 24 h or 48 h treatment with 20 ng/ml or 40 ng/ml of IL-6. Taken together, these results indicated that the antiviral effect of IL-6 is not due to the disruption of capsid integrity as the TNF-α [11]. On the other hand, our results suggest that IL-6 may prevent the formation of genome-containing nucleocapsids similar to interferons [10]).

### The antiviral effect of IL-6 was not mediated through induction of interferons

**Figure 6:**
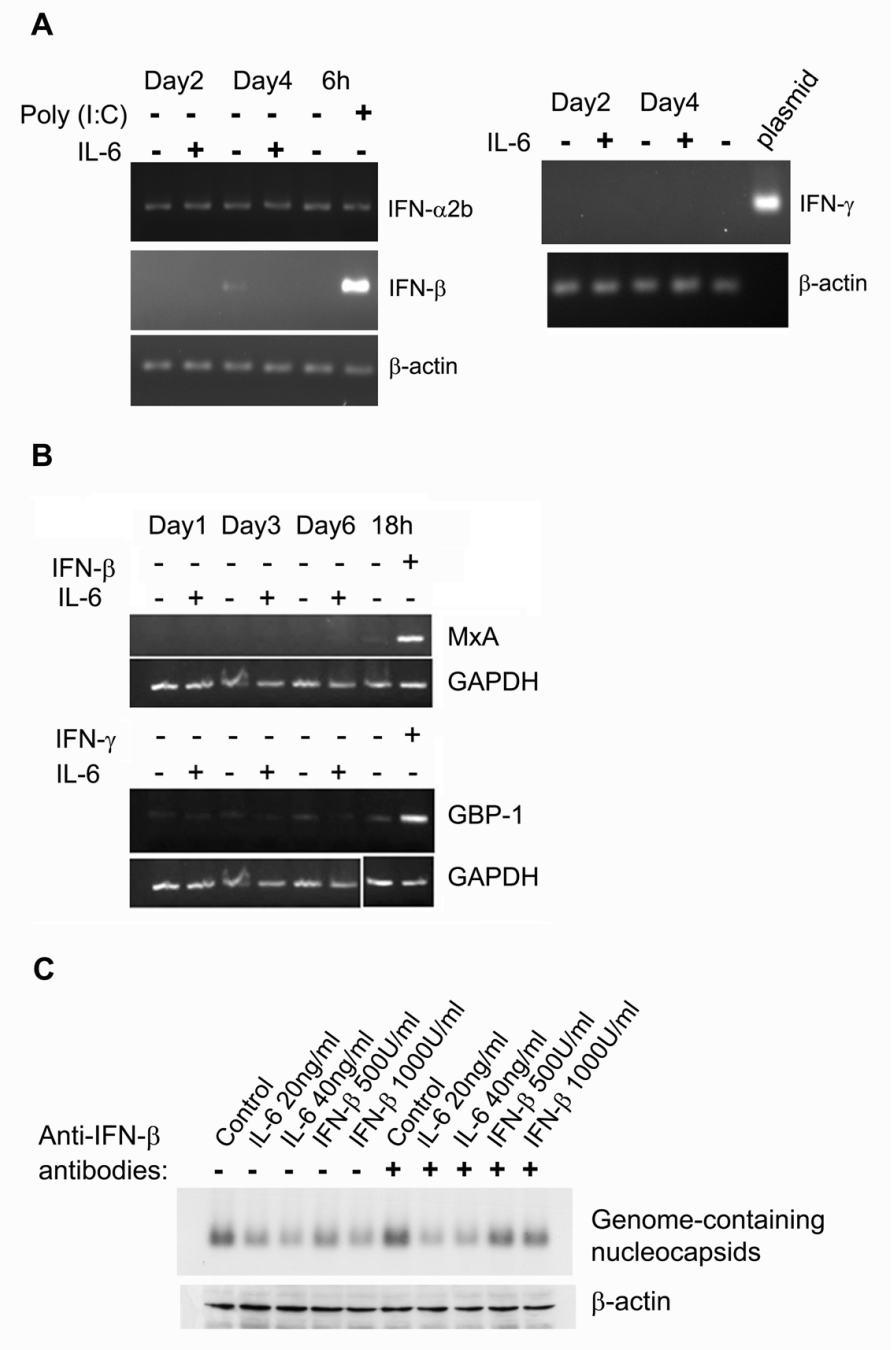
**The antiviral effect of IL-6 on HBV replication is not mediated through induction of IFNs**. (A) Confluent 1.3ES2 cells were treated with or without 20 ng/ml of IL-6 for 2 day (day2) or 4 days (day4). Total RNA of each sample was extracted for the detection of IFN-α2b, IFN-β, IFN-γ and β-actin by RT-PCR analysis. Cells were transfected with 20 μg poly (I:C) for 6 h and served as the positive control for the induction of IFN-α2b and IFN-β simultaneously. Plasmid containing IFN-γ gene was served as the positive control for the detection of IFN-γ. (B) Confluent 1.3ES2 cells were treated with or without 20 ng/ml of IL-6 for 1 day (day1), 3 days (day3) or 6 days (day6). Total RNA of each sample was extracted for the detection of GBP-1 (target gene for IFN-γ), MxA (target gene for type I IFN) and GAPDH by RT-PCR analysis. Cells were treated with IFN-β or IFN-γ for 18 h and served as the positive control for the induction of GBP-1 and MxA genes. (C) Confluent 1.3ES2 cells were treated with various concentration of IL-6 or IFN-β for 4 days in the absence or presence of anti-IFN-β polyclonal antibody (400 U/ml). The level of HBV genome-containing nucleocapsids was measured as mentioned above. The expression level of β-actin was used as an internal control for sample loading.

IFN-α/β and IFN-γ have been reported to noncytopathically suppress HBV replication through the prevention of HBV genome-containing nucleocapsids production in cell culture systems [10]. To assess whether interferons were involved in the antiviral effect of IL-6 on HBV replication, the induction of IFN-α/β and IFN-γ was measured in the 1.3ES2 cells after IL-6 treatment. Using RT-PCR analysis, we found that the expression of IFN-α2b, IFN-β and IFN-γ was not induced after 6 days of treatment with IL-6 (Fig. 6A). Similarly, the expression of MxA (the target gene for type I IFN) and GBP-1 (the target gene for IFN-γ) was not induced under the same condition (Fig. 6B). Furthermore, anti-IFN-β polyclonal antibody was added to the culture medium to block the endogenous IFN activity and examine its effect on the suppression of HBV replication induced by IL-6. As shown in Fig. 6C, the suppressive effect of IL-6 on the level of HBV genome-containing nucleocapsids was not affected by the presence of anti-IFN-β antibody. On the other hand, IFN-β-mediated antiviral effect was blocked by the anti-IFN-β antibody. In conclusion, our results clearly indicated that IFN-α/β and IFN-γ did not participate in the IL-6-induced suppression of HBV replication

### IL-6 prevents the accumulation of HBV cccDNA

**Figure 7:**
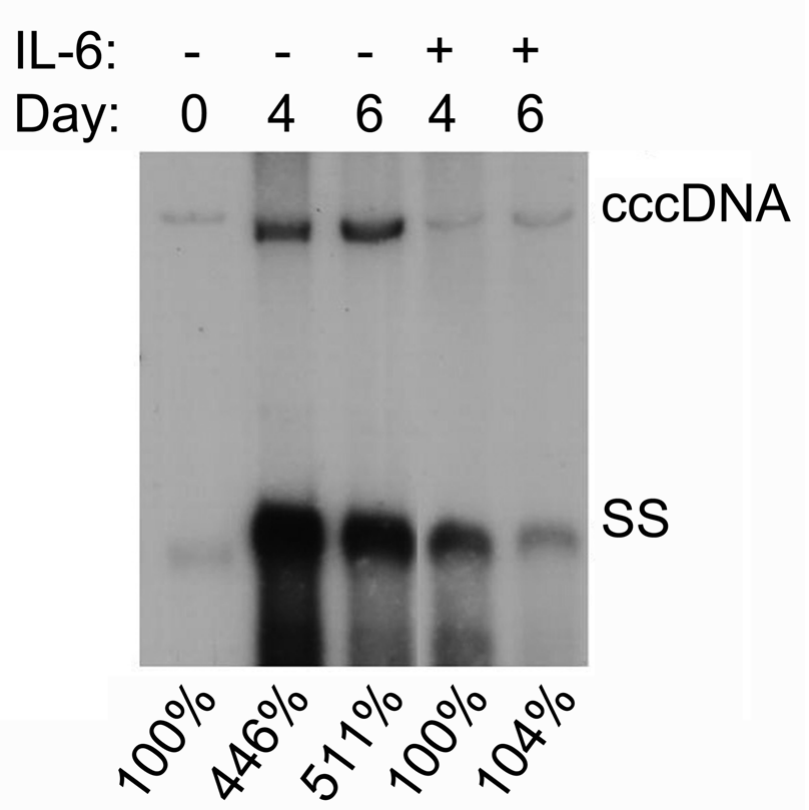
**IL-6 decreases the formation of HBV cccDNA**. Confluent cells (day0) were treated with or without 20 ng/ml of IL-6 for 4 days (day4) or 6 days (day6) and subjected to Hirt extraction to prepare HBV cccDNA. The Hirt extract was thermally denatured at 85°C for 5 min, digested with EcoRI and analyzed by Southern blotting using an HBV-specific probe. Bands corresponding to HBV cccDNA and HBV single-stranded DNA (SS) are indicated. The signals were quantified by densitometry analysis and expressed as percentage of the control cells to indicate the IL-6 effect on the accumulation of HBV cccDNA.

HBV cccDNA plays a pivotal role in the life cycle of HBV replication and maintenance of HBV persistent infection. The observation that IL-6 effectively decreased the level of HBV replicative intermediates raised the possibility that IL-6 might also affect the formation of HBV cccDNA. To examine the IL-6 effect on the amount of cccDNA, viral nucleic acid was prepared by Hirt extraction after 4 days or 6 days treatment with or without IL-6. The replicative intermediates including relax-circular and duplex-linear form DNA, but not cccDNA, were denatured into single-stranded DNA after melting at 85°C for 5 min. Subsequent EcoRI digestion linearized the intact cccDNA and the DNA's mobility was shifted from cccDNA to the position of the duplex-linear form DNA (3.2 kb). Southern blot analysis demonstrated that the amount of HBV cccDNA was significant reduced in the presence of IL-6, as compared with the mock-control cells (Fig. 7). However, the amount of cccDNA after 6 days of IL-6 treatment was similar to the pre-existing level of cccDNA of 1.3ES2 cells at day 0. These results indicated that IL-6 was able to effectively prevent the accumulation of cccDNA, but was not able to decrease the existing pool of cccDNA.

## Discussion

In this study, we found that IL-6 suppressed HBV replication in an HBV-replicating cell line. The inhibitory effect was not a result of cell apoptosis since IL-6 treatment increased cell proliferation. We also demonstrated that IL-6 exerted its inhibitory effect through a combination of two different mechanisms which included a moderate reduction of HBV transcripts/core protein coordinately and a marked decrease in the level of HBV genome-containing nucleocapsids. Furthermore, IL-6 also prevents the accumulation of HBV cccDNA. IL-6 has recently been found to suppress HCV RNA replication and protein expression in liver cells [25]. This result coincides with our findings and supports the suggestion that IL-6 is an antiviral cytokine during the progression of chronic hepatitis.

The pathogenesis of HBV-induced liver diseases involves complicated mechanisms related to viral replication and the body's immune responses against HBV infection [26], including HBV-specific cell-mediated immunity (CMI) and inflammatory cytokines. The CMI responses are essential for the resolution of HBV infection, but several studies have indicated that cytokines are involved in the noncytopathic suppression of virus replication [5]. Of significance to the present study is that the levels of many cytokines (including IL-18, IFNs, TNF-α, IL-4 and TGF-β1) are elevated during hepatitis progression, and have been demonstrated to exhibit antiviral activity in both transgenic mice and cell culture systems [8-10,12,13,24]. Interestingly, the levels of serum IL-6 were reported to represent the best marker of HBV-related clinical progression as compared with IL-10, IL-12 and IFN-γ [20]. The inhibitory mechanisms of the cytokines can be generally divided into two major classes. The first class, which includes IL-4 and TGF-β1, suppresses viral gene expression and subsequently blocks HBV replication. IL-4 suppresses HBV replication through down-regulation of C/EBPα, while TGF-β1 reduces HBV replication through transcriptional inhibition of pregenomic RNA. Cytokines of the second class exert their antiviral effect by either destabilizing viral genome-containing capsids or preventing their assembly. Types I and II IFN activate hepatocellular processes, which prevent the formation of viral genome-containing capsids and subsequently inhibit HBV replication [10], whereas the inhibitory effect of TNF-α involves the destabilization of viral nucleocapsids [27]. In this study, we demonstrated that IL-6 exerted its antiviral effect through a moderate reduction of viral transcripts similar to IL-4 and TGF-β1 and a more dramatic reduction of viral genome-containing nucleocapsids. These results indicate that the inhibitory mechanism of IL-6 in HBV replication is a combination of both classes of cytokines.

The IL-6 effect on the reduction of HBV genome-containing nucleocapsids may be similar to interferons and mediated through the prevention of the formation of genome-containing nucleocapsids since there is no evidence of the disruption of capsid integrity after IL-6 treatment. The expression levels of IFNs and IFN target genes were not induced during the IL-6 treatment. These results indicate that IL-6 induced suppression of HBV replication is not mediated through the induction of IFNs.

The formation of HBV genome-containing nucleocapsids involves very complicated processes. These processes include dimerization of core protein and multimer association, binding of RNA polymerase to ε structure of pregenomic RNA (pgRNA) and packaging of pgRNA, viral polymerase and cellular factors such as heat shock proteins into capsids [28,29]. The IL-6 effect on the prevention of the formation of viral genome-containing nucleocapsids could occur at any step of these processes. Similar to the inhibitory effect of IFNs, the exact mechanism involved in the antiviral effect of IL-6 remains to be further investigated.

## Competing interests

The authors declare that they have no competing interests.

## Authors' contributions

TMK performed the major experiments and analyzed the data. CPH participated in the design of the study and data interpretation. YLC and MHH participated in part of the experiments. KSJ established the 1.3ES2 cell line. CCTL participated in the data interpretation and manuscript improvement. MLC and CC designed the experiments, interpreted the data and wrote the manuscript. All authors read and approved the final manuscript.
